# Peroxide of Hydrogen as Antiseptic and Disinfectant

**Published:** 1890-02

**Authors:** 


C. T. KINGZETT, F.I.C., F.C.S., in his book entitled "Nature's Hygiene," London, Bailliere, Tindall & Cox, describes the uses of peroxide of hydrogen as antiseptic and disinfectant, and dispels much of the mystery surrounding the name "Sanitas." It appears, Mr. Kingzett found reason to believe, that plants do not produce ozone, but peroxide of hydrogen, and undertook a series of experiments to clear up this point in natural chemistry. He accordingly submitted to the influence of atmospheric air and oxygen a large number of oils, such as oils of turpentine, caraway, bergamot, eucalyptus and juniper. He found that oxygen was absorbed readily by all these substances, and in the case of turpentine to an enormous extent. Mr. Kingzett next found that when turpentine was exposed to a current of air the presence of water, especially at summer temperatures, oxygen was absorbed, the oil became oxidized into a compound, unstable in the presence of water, and splitting up thereby into peroxide of hydrogen, champhoric acid, etc. The result of these experiments was to convince the investigator, firstly, that the hygienic influence common to groves of pine and blue gum-trees is to be attributed to the constant evolution of peroxide of hydrogen, soluble camphor, camphoric acid, thymol, and certain other camphoraceous bodies, caused by volatilization and oxidation of naturally secreted oils; and secondly, that this depended upon the atmospheric oxidation of the "terpene," or principle of turpentine common to all, and most powerful in the ordinary turpentine of commerce derived from the pine.

Having then arrived at the facts that the healthy atmosphere of a pine wood or an eucalyptus forest is due to the presence of peroxide of hydrogen and camphoraceous substances produced by the atmospheric oxidation of the essential oils secreted by those trees, and that these valuable natural purifiers can be produced from common turpentine, Mr. Kingzett manufactured them in such quantities as would make them practically available as antiseptics and disinfectants—in short, to imitate the effect produced on a grand scale in nature's own laboratory. The disinfectant properties of peroxide of hydrogen has long been known to the profession; but its mode of preparation has been too expensive. His discovery consists in the antiseptic qualities of peroxide of hydrogen, and the invention of a method of preparation which brings the healthful principles within the reach of the public.

By exposing turpentine floating on water to a hot blast, a solution, composed of peroxide of hydrogen, camphoric acid, camphor, thymol, etc., is produced, and an oil containing camphoric peroxide. To these products the name "Sanitas" has been given.

It is evident that the disinfectant depends largely upon the camphoric peroxide. Recent investigations by M. Miguel, of the Observatorie de Montsouris, appear to establish the fact that peroxide of hydrogen is one of the most potent destroyers of bacterial life. He states "that careful comparative tests prove it to be sixty times as powerful as carbolic acid, twenty times as strong as salicylic acid, and 40 per cent. more potent than the solution of bichloride of mercury."—*Dietetic Gazette.*

